# Supplementary material for: Modeling glioblastoma heterogeneity as a dynamic network of cell states
Source: Mol Syst Biol. 2021 Sep 16;17(9):e10105. doi: 10.15252/msb.202010105 (PMC8444284; doi:10.15252/msb.202010105)
Supplement: Supplementary file 6 — Source Data for Figure 5 [file MSB-17-e10105-s004.zip › Figure5A_sourcedata/GSEA_3071/hallmarks_stateVI.GseaPreranked.1623742557031/HALLMARK_P53_PATHWAY.html]

Details for gene set HALLMARK\_P53\_PATHWAY[GSEA]

|  || Dataset | state63071 |
| Phenotype | NoPhenotypeAvailable |
| Upregulated in class | na\_neg |
| GeneSet | HALLMARK\_P53\_PATHWAY |
| Enrichment Score (ES) | -0.20112678 |
| Normalized Enrichment Score (NES) | -0.9583564 |
| Nominal p-value | 0.5328798 |
| FDR q-value | 0.751633 |
| FWER p-Value | 1.0 |
Table: GSEA Results Summary

  

Fig 1: Enrichment plot: HALLMARK\_P53\_PATHWAY      
 Profile of the Running ES Score & Positions of GeneSet Members on the Rank Ordered List

  

| PROBE | GENE SYMBOL | GENE\_TITLE | RANK IN GENE LIST | RANK METRIC SCORE | RUNNING ES | CORE ENRICHMENT || 1 | DDIT3 |  |  | 14 | 1.086 | 0.0237 | No |
| 2 | PERP |  |  | 27 | 0.969 | 0.0450 | No |
| 3 | ADA |  |  | 63 | 0.842 | 0.0566 | No |
| 4 | PPP1R15A |  |  | 98 | 0.738 | 0.0658 | No |
| 5 | TM4SF1 |  |  | 123 | 0.703 | 0.0769 | No |
| 6 | SPHK1 |  |  | 141 | 0.677 | 0.0894 | No |
| 7 | HBEGF |  |  | 172 | 0.627 | 0.0969 | No |
| 8 | GADD45A |  |  | 200 | 0.603 | 0.1047 | No |
| 9 | UPP1 |  |  | 218 | 0.590 | 0.1149 | No |
| 10 | TAX1BP3 |  |  | 275 | 0.551 | 0.1132 | No |
| 11 | PROCR |  |  | 314 | 0.532 | 0.1161 | No |
| 12 | JUN |  |  | 321 | 0.527 | 0.1278 | No |
| 13 | MKNK2 |  |  | 326 | 0.524 | 0.1400 | No |
| 14 | PLK2 |  |  | 363 | 0.505 | 0.1427 | No |
| 15 | TRIB3 |  |  | 387 | 0.495 | 0.1488 | No |
| 16 | TXNIP |  |  | 402 | 0.487 | 0.1573 | No |
| 17 | RACK1 |  |  | 407 | 0.486 | 0.1685 | No |
| 18 | ATF3 |  |  | 416 | 0.483 | 0.1786 | No |
| 19 | PTPN14 |  |  | 456 | 0.469 | 0.1796 | No |
| 20 | SOCS1 |  |  | 489 | 0.458 | 0.1822 | No |
| 21 | SLC3A2 |  |  | 559 | 0.437 | 0.1740 | No |
| 22 | RPL18 |  |  | 564 | 0.436 | 0.1839 | No |
| 23 | IRAK1 |  |  | 665 | 0.408 | 0.1663 | No |
| 24 | MAPKAPK3 |  |  | 785 | 0.379 | 0.1425 | No |
| 25 | RAP2B |  |  | 802 | 0.376 | 0.1476 | No |
| 26 | TPRKB |  |  | 869 | 0.365 | 0.1384 | No |
| 27 | RPL36 |  |  | 878 | 0.364 | 0.1454 | No |
| 28 | BMP2 |  |  | 940 | 0.354 | 0.1373 | No |
| 29 | IER5 |  |  | 950 | 0.353 | 0.1437 | No |
| 30 | CCND2 |  |  | 1064 | 0.335 | 0.1206 | No |
| 31 | RPS27L |  |  | 1136 | 0.325 | 0.1089 | No |
| 32 | RHBDF2 |  |  | 1151 | 0.323 | 0.1132 | No |
| 33 | RCHY1 |  |  | 1175 | 0.319 | 0.1149 | No |
| 34 | RPS12 |  |  | 1219 | 0.313 | 0.1108 | No |
| 35 | BAK1 |  |  | 1249 | 0.310 | 0.1105 | No |
| 36 | JAG2 |  |  | 1281 | 0.306 | 0.1096 | No |
| 37 | BAIAP2 |  |  | 1490 | 0.281 | 0.0584 | No |
| 38 | ZNF365 |  |  | 1610 | 0.268 | 0.0318 | No |
| 39 | CCNK |  |  | 1633 | 0.265 | 0.0324 | No |
| 40 | RRP8 |  |  | 1687 | 0.261 | 0.0241 | No |
| 41 | PLK3 |  |  | 1710 | 0.259 | 0.0245 | No |
| 42 | CDKN2B |  |  | 1796 | 0.251 | 0.0071 | No |
| 43 | LIF |  |  | 1804 | 0.250 | 0.0115 | No |
| 44 | CTSD |  |  | 1862 | -0.259 | 0.0021 | No |
| 45 | SAT1 |  |  | 1952 | -0.271 | -0.0160 | No |
| 46 | KRT17 |  |  | 2147 | -0.304 | -0.0627 | No |
| 47 | TSC22D1 |  |  | 2193 | -0.313 | -0.0674 | No |
| 48 | TRAF4 |  |  | 2263 | -0.326 | -0.0784 | No |
| 49 | MXD4 |  |  | 2312 | -0.335 | -0.0834 | No |
| 50 | EPHX1 |  |  | 2327 | -0.339 | -0.0787 | No |
| 51 | BAX |  |  | 2397 | -0.353 | -0.0891 | No |
| 52 | MDM2 |  |  | 2419 | -0.357 | -0.0859 | No |
| 53 | ABAT |  |  | 2438 | -0.365 | -0.0817 | No |
| 54 | F2R |  |  | 2478 | -0.372 | -0.0831 | No |
| 55 | IP6K2 |  |  | 2491 | -0.376 | -0.0769 | No |
| 56 | ZMAT3 |  |  | 2590 | -0.399 | -0.0943 | No |
| 57 | CYFIP2 |  |  | 2619 | -0.404 | -0.0919 | No |
| 58 | PRMT2 |  |  | 2839 | -0.470 | -0.1414 | No |
| 59 | FOXO3 |  |  | 3053 | -0.564 | -0.1868 | Yes |
| 60 | CD81 |  |  | 3056 | -0.566 | -0.1730 | Yes |
| 61 | WWP1 |  |  | 3153 | -0.621 | -0.1841 | Yes |
| 62 | TM7SF3 |  |  | 3182 | -0.644 | -0.1756 | Yes |
| 63 | NOTCH1 |  |  | 3192 | -0.657 | -0.1614 | Yes |
| 64 | RB1 |  |  | 3229 | -0.697 | -0.1538 | Yes |
| 65 | RGS16 |  |  | 3304 | -0.765 | -0.1551 | Yes |
| 66 | FBXW7 |  |  | 3315 | -0.779 | -0.1381 | Yes |
| 67 | NUPR1 |  |  | 3366 | -0.835 | -0.1309 | Yes |
| 68 | CCP110 |  |  | 3376 | -0.856 | -0.1116 | Yes |
| 69 | BTG2 |  |  | 3377 | -0.857 | -0.0899 | Yes |
| 70 | CD82 |  |  | 3408 | -0.912 | -0.0751 | Yes |
| 71 | CDH13 |  |  | 3410 | -0.913 | -0.0521 | Yes |
| 72 | PCNA |  |  | 3415 | -0.922 | -0.0298 | Yes |
| 73 | FOS |  |  | 3476 | -1.056 | -0.0198 | Yes |
| 74 | NINJ1 |  |  | 3500 | -1.117 | 0.0021 | Yes |
| 75 | APP |  |  | 3562 | -1.427 | 0.0213 | Yes |
Table: GSEA details [plain text format]

  

Fig 2: HALLMARK\_P53\_PATHWAY: Random ES distribution      
 Gene set null distribution of ES for **HALLMARK\_P53\_PATHWAY**
